# Different Analgesia Techniques for Postoperative Pain in Children Undergoing Abdominal Surgery for Intractable Constipation: A Retrospective Cohort Study in a Single Tertiary Children’s Hospital

**DOI:** 10.3390/jcm13020349

**Published:** 2024-01-08

**Authors:** Manouk Admiraal, Fleur A. E. van der Burg, Henning Hermanns, Jeroen Hermanides, Markus W. Hollmann, Marc A. Benninga, Justin de Jong, Ramon R. Gorter, Markus F. Stevens

**Affiliations:** 1 Department of Anesthesiology, Amsterdam UMC, University of Amsterdam, Meibergdreef 9, 1105 AZ Amsterdam, The Netherlands; m.admiraal1@amsterdamumc.nl (M.A.); fvanderburg@spaarnegasthuis.nl (F.A.E.v.d.B.); j.hermanides@amsterdamumc.nl (J.H.); m.w.hollmann@amsterdamumc.nl (M.W.H.); m.f.stevens@amsterdamumc.nl (M.F.S.); 2 Department of Pediatric Gastroenterology, Emma Children’s Hospital, Amsterdam UMC, University of Amsterdam, Meibergdreef 9, 1105 AZ Amsterdam, The Netherlands; m.a.benninga@amsterdamumc.nl; 3 Department of Pediatric Surgery, Emma Children’s Hospital, Amsterdam UMC, University of Amsterdam and Vrije Universiteit Amsterdam, Meibergdreef 9, 1105 AZ Amsterdam, The Netherlands; j.r.dejong@amsterdamumc.nl (J.d.J.); rr.gorter@amsterdamumc.nl (R.R.G.)

**Keywords:** child, constipation, surgical procedures, operative, pain, postoperative, pain management

## Abstract

Functional constipation in the pediatric population is a prevalent issue that is usually well managed. However, in rare cases, conservative treatment fails, and surgical intervention is necessary. This retrospective cohort study aimed to describe and compare different perioperative analgesic techniques in children undergoing major abdominal surgery for intractable constipation. Conducted between 2011 and 2021, this study enrolled patients under 18 years old who underwent initial major abdominal surgery for intractable constipation (i.e., creation of ostomy or subtotal colectomy). Patients were categorized according to the perioperative analgesic technique (i.e., systemic, neuraxial, or truncal block). Of 65 patients, 46 (70.8%) were female, and the median age was 13.5 [8.8–16.1] years during initial major abdominal surgery. Systemic analgesia was used in 43 (66.2%), neuraxial in 17 (26.2%), and truncal blocks in 5 (7.7%) of the surgeries. Patients with neuraxial analgesia reported less postoperative pain (median [interquartile range] numeric rating scale (NRS) 2.0 [0–4.0]), compared to systemic analgesia (5.0 [2.0–7.0], *p* < 0.001) and to truncal blocks (5.0 [3.0–6.5], *p* < 0.001). In this preliminary investigation, neuraxial analgesia appears to be the most effective approach to reducing acute postoperative pain in pediatric patients undergoing major abdominal surgery for intractable functional constipation. However, well-designed studies are warranted.

## 1. Introduction

Constipation in children is a complex and challenging problem that can have a significant impact on the health-related quality of life of patients, parents or guardians, and healthcare providers [1]. The most common cause of constipation is functional constipation, with a reported prevalence of 9.5% in infants worldwide [2]. Functional constipation can be diagnosed according to the Rome IV criteria when an infant has ≥2 of the following criteria for ≥1 month: two or fewer defecations per week, history of excessive stool retention, history of painful or hard bowel movements, history of large-diameter stools, presence of a large fecal mass in the rectum. Additional criteria can be employed in children who have been toilet-trained: at least one episode/week of incontinence and history of large-diameter stools that may obstruct the toilet [3].

Constipation is commonly managed through a step-up approach involving non-pharmacological methods, such as education, toilet training, as well as pharmacological treatments such as oral laxatives [4]. If these measures are not effective, enemas or transanal irrigation may be employed [4]. However, despite optimal oral and rectal treatment, some patients continue to experience symptoms that can negatively impact their psychosocial wellbeing. In such cases, minor abdominal surgeries such as appendicostomy or cecostomy may be considered [5]. Lastly, in rare cases where the patients do not respond to any of these treatments, major abdominal surgery such as ileostomy, colostomy, sigmoid resection, or even (sub)total colectomy may be necessary. The outcomes of these surgeries for intractable constipation vary and are dependent on individual factors [6].

Pediatric patients with intractable functional constipation pose a potentially challenging population for perioperative pain management. Studies indicate that children who frequently visit the emergency department due to constipation report an average abdominal pain score 6 out of 10 with a standard deviation of 4 [7]. This persistent pain can lead to central sensitization and pain hypersensitivity, which could impair effective perioperative pain management and increase the likelihood of transitioning to chronic post-surgical pain [8,9]. Second, the use of opioids, a commonly used pain relief treatment, may exacerbate constipation in this population [10]. As an alternative, regional techniques have been suggested, which have been demonstrated to be safe in children [11]. Thirdly, this population has a higher incidence of neuropsychiatric disorders, which can negatively impact postoperative pain management [12,13,14]. Hence, managing perioperative pain in pediatric patients with intractable functional constipation requires careful consideration of these challenges.

Despite the complexity of this patient population, there has been a lack of research investigating the most effective approach to managing postoperative pain in children with intractable functional constipation undergoing abdominal surgery. Therefore, the aim of this study was to explore the effectiveness of different analgesic techniques in reducing pain for patients with intractable constipation undergoing surgery.

## 2. Materials and Methods

### 2.1. Study Design and Participants

This retrospective exploratory study was conducted in a tertiary center for children with defecation disorders in the Netherlands. Patients under the age of 18 years who underwent their first major abdominal surgery for intractable functional constipation between 2011 and 2021 were considered eligible for inclusion in this study. Intractable functional constipation was characterized by persistent symptoms that negatively affected psychosocial functioning despite receiving optimal intensive and long-standing oral and/or rectal treatment for constipation [15]. The surgical interventions for intractable constipation included procedures such as ileostomy, colostomy, sigmoid resection, or a subtotal colectomy [16]. The term “initial surgery” referred to the first major surgical procedure performed within the abdominal region. The time span of 2011–2021 was chosen because there was limited available information regarding patients and conducted operations before this period. Patients who only underwent minor surgery, such as sacral nerve stimulation, anal sphincter botulin toxin injections, appendicostomy, cecostomy, gastrostomy, or jejunostomy, were excluded. Additionally, patients with organic causes of constipation (i.e., Hirschprung’s disease, pediatric intestinal pseudo-obstruction, anorectal malformations) and patients lacking any postoperative numeric rating scale (NRS) scores were excluded.

This study was designated as exempt from oversight by the Institutional Review Board (W21_164 # 21.179) This determination was made by the accredited medical research ethics committee in the Netherlands on 6 April 2021. All eligible patients or their parents were contacted by their surgeon to object or consent to the use of their data. This study adhered to the Declaration of Helsinki and to the Strengthening the Reporting of Observational Studies in Epidemiology (STROBE) guidelines for reporting [17].

### 2.2. Procedures

The decision to perform surgery was made by a multidisciplinary team consisting of a pediatric gastroenterologist, pediatric surgeons, anesthesiologists, medical psychologists, pediatric psychiatrist, nurse specialists, dieticians, a pelvic floor physiotherapist, and a (social) pediatrician. This team carefully evaluated the patients through a diagnostic workup, which included ruling out organic causes of constipation, analyzing their psychological status, and exploring non-surgical treatment options. Multidisciplinary team meetings were conducted every two weeks. Before surgery, the individualized analgesic strategy was discussed between a pediatric anesthesiologist, the parents, and, if possible, the pediatric patient.

Before starting surgery, the preferred method for anesthesia induction was determined on an individualized basis, considering the options of intravenous administration or utilizing a vapor hood. When opting for intravenous induction, patients were prepped pre-operatively with a plaster containing eutectic mixture of lidocaine (EMLA) cream. This facilitated local anesthesia for the pediatric patient during the subsequent insertion of an intravenous access. Alternatively, the selection of the vapor hood involved the administration of sevoflurane to induce anesthesia, followed by the insertion of an intravenous line after the patient had entered a state of unconsciousness. Following either method, the appropriate soporific agent was administered, and the patient underwent intubation using an age-appropriate tube. Subsequently, patients were equipped with a urinary catheter and nasogastric tube as part of the procedural protocol. Anesthesia was administered using propofol, sevoflurane, or a combination of both agents.

Furthermore, systemic analgesia was administered to all patients during the surgical procedure, based on the preference of the attending anesthesiologist. In recent years, there has been a growing emphasis on opioid-sparing, multimodal analgesia approaches in our hospital. Each patient was administered standard doses of paracetamol at 15 mg/kg, dexamethasone at 0.1 mg/kg, and metamizole at 15 mg/kg as per consultation with the surgeon. Furthermore, opioids were administered, and when deemed necessary, clonidine, esketamine, and lidocaine were given. Epidural, caudal, and truncal blocks were performed intraoperatively while the patient was under anesthesia. The anesthesiologist employed an ultrasound-guided nerve identification technique to perform truncal blocks.

When feasible, the extubation of the patients occurred in the operating room before transferring them to the post-anesthetic care unit (PACU). Evaluating a patient’s discharge from the postoperative recovery unit involved assessing multiple criteria, including vital sign stability, awakening and consciousness, pain management, control of nausea and vomiting, mobility and motor skills, as well as hydration and urination [18].

In the hospital ward, patients were administered paracetamol, metamizole, or non-steroidal anti-inflammatory drugs (NSAIDs). If necessary, opioids were provided orally for mild pain or intravenously for moderate to severe pain. Patients under the age of six years received continuous intravenous morphine, whereas those aged six and above were equipped with an intravenous morphine patient-controlled analgesia pump. Consultation with the Acute Pain Service (APS) post-surgery occurred in instances requiring specialized pain management, encompassing scenarios such as complex pain management, the utilization of advanced pain techniques, or specific conditions.

### 2.3. Data Collection and Measurements

Patients were categorized into three groups according to the used analgesic technique during surgery: (1) systemic analgesia (i.e., solely utilizing systemic administration of analgesics), (2) the neuraxial technique (i.e., continuous epidural analgesia or caudal/epidural blocks), (3) truncal blocks (i.e., a truncal block such as a transversus abdominis plane block, quadratus lumborum block, or rectus sheath block).

Pediatric nurses trained in evaluating age-adapted pain measurement assessed pain using the numeric rating scale (NRS) score, monitoring it a minimum of three times daily within the initial 72 h period post-surgery, according to the hospital protocol. The intraoperative use of opioids was analyzed via its conversion into intravenous morphine milligram equivalents (MME) (Appendix A) [19]. The MME was divided by the weight and duration of anesthesia to calculate the intravenous MME/kg/hour. The duration of the PACU stay was measured in minutes, whereas the hospital admission length was measured in days. Rescue interventions during the postoperative period due to insufficient pain management were defined as systematic analgesic or regional analgesic interventions, such as neuraxial or truncal blocks. Adverse events related to the analgesic technique used, such as postoperative nausea and vomiting (PONV), the systemic toxicity of local anesthetics, extended motor block, signs of infections associated with neuraxial catheters, and neurologic damage, were registered.

The data were collected in Castor Electronic Data Capture (Ciwit B.V., v. 1.5, Amsterdam, the Netherlands), which is a good clinical practice compliant anonymized database, from electronic patient records.

### 2.4. Outcomes

The primary outcome was the difference in pain, as measured by the NRS score, between the different analgesic techniques (i.e., systemic analgesia, neuraxial, or truncal block) during the first 24 h after surgery.

The secondary outcomes included differences in pain, as measured by the NRS score, between the different analgesic techniques (i.e., systemic analgesia, neuraxial, or truncal block) during the second until fifth 24 h after surgery; the length of stay (both PACU and hospital admission); the number of patients who were transferred to the PACU for rescue interventions because of inadequate pain management; and the number of adverse events related to the analgesic technique.

### 2.5. Statistical Analysis

To describe nominal or ordinal variables, percentages and frequencies were used. Mean and standard deviations (SD) were used to report normally distributed continuous data, whereas median and interquartile range (IQR) were used for non-normally distributed continuous data. Normality was assessed using both visual inspection and the Shapiro–Wilk test. To statistically test for differences between groups, the Kruskal–Wallis test was employed for non-parametric continuous data. If the Kruskal–Wallis test turned out to be significant, the Dunn test, also called the Bonferroni–Dunn test, was performed for post hoc testing between each independent group.

Statistical uncertainties were quantified with two-sided 95% confidence intervals. A two-sided *p*-value < 0.05 was considered statistically significant. All statistical analysis were performed using RStudio (Posit, Boston, MA, USA, Affero General Public License v.3).

## 3. Results

### 3.1. Baseline Characteristics

**Figure 1:**
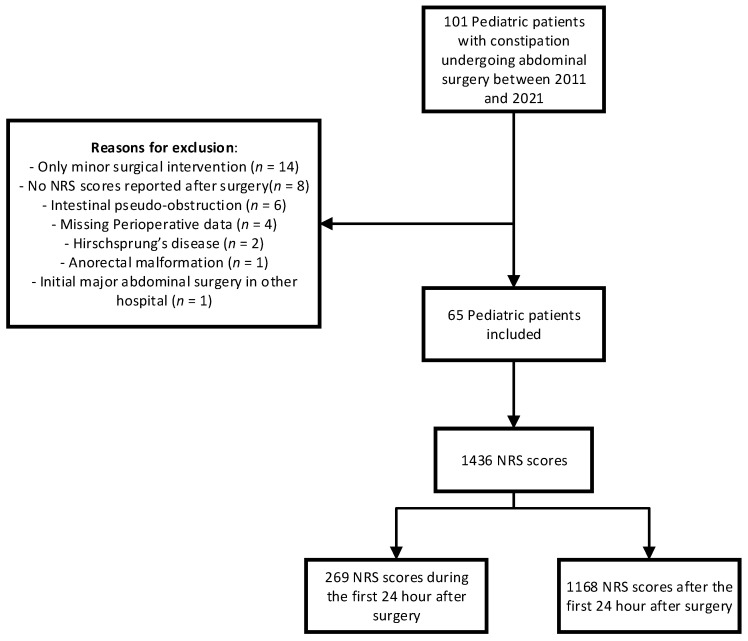
Flow diagram: NRS = numeric rating scale.

In total, 101 pediatric patients with constipation undergoing abdominal surgery between 2011 and 2021 were identified, out of which 65 patients were included in the study. Figure 1 presents a flow diagram illustrating various reasons for exclusion, such as an organic cause for constipation, the performance of minor surgical procedures only, or missing data.

The distribution of surgeries over time is shown in Appendix A. Of the total patients, 46 (70.8%) patients were female, and the median [IQR] age during initial major abdominal surgery was 13.5 [8.8, 16.1] years. The age of onset of constipation was 2.0 [0, 7.0] years. Patients often had a history of psychological conditions or were affected by behavioral problems; see Table 1.

### 3.2. Anesthetic and Surgical Characteristics

Of the 65 initial major abdominal surgeries conducted, 40 involved an ileostomy, 12 a colostomy, 15 a sigmoidectomy, and 5 patients underwent subtotal colectomy. The laparoscopic technique was used in 45 (69.2%) patients, open surgery in 8 (12.3%) patients, and a laparoscopic hand-assisted technique was utilized in 12 (18.5%) patients. The used analgesic technique during surgery was systemic analgesia in 43 (66.2%) surgeries, neuraxial analgesia in 17 (26.2%) surgeries, and truncal blocks in 5 (7.7%) surgeries. Of the patients who received truncal blocks, three (60.0%) patients received a transversus abdominis plane block, one (20.0%) patient a quadratus lumborum block, and one (20.0%) patient a rectus sheath block. Table 2 displays the perioperative characteristics, including the specific systemic analgesia utilized.

### 3.3. Primary Outcome

During the first 24 h after surgery, 269 NRS scores were registered, and a difference in NRS scores was observed between the analgesic groups (*p* < 0.001). Patients with neuraxial analgesia reported lower postoperative pain scores (median [IQR] NRS 2.0 [0, 4.0]) compared to patients with systemic analgesia only (5.0 [2.0, 7.0], *p* < 0.001) and compared to truncal blocks (5.0 [3.0, 6.5], *p* < 0.001). No difference in pain scores was found between systemic analgesia only and truncal blocks, *p* = 0.971, Table 3.

### 3.4. Secondary Outcomes

During the second and third 24 h after surgery, 187 and 190 scores were registered, respectively. In both periods, the NRS scores differed between groups, with the lowest reported pain scores in patients with neuraxial analgesia compared to patients with systemic analgesia only and compared to truncal blocks. No difference was found between systemic analgesia only and truncal blocks; see Table 3. During the fourth and fifth 24 h after surgery, the NRS scores were similar between groups.

The length of stay was comparable in the PACU (median [IQR] 179.5 [143.5, 252.0] vs. 175.5 [144.0, 227.2] vs. 190.0 [185.0, 210.0] minutes, *p* = 0.911) as was hospital admission (median [IQR] 10.0 [6.0, 12.0] vs. 10.0 [7.0, 17.0] vs. 11.0 [10.0, 12.0] days, *p* = 0.389) for systematic analgesia, neuraxial blocks, and truncal blocks, respectively. During the postoperative phase, four patients were transferred from the ward to the PACU for additional pain management. Among these patients, three patients had received solely systemic analgesia, while one patient had received a neuraxial blockade during surgery. In order to manage the inadequately controlled pain, morphine titration was utilized and found to be successful in one out of four patients. Among the remaining three patients, truncal blocks were administered, but only one of them experienced relief from pain. Following this, one of the two patients with persistent pain received an epidural catheter, while the other received an additional truncal blockade combined with an infusion of esketamine. No adverse events related to the anesthetic procedures were registered.

## 4. Discussion

In this study, neuraxial analgesia appeared to be superior in reducing pain at the first 72 h after surgery. However, besides its statistical significance, it is also worth investigating its clinically relevant effectiveness. While the precise threshold of a “clinically meaningful” difference in pain is vividly discussed, a difference of more than two points is almost universally accepted as a clinically relevant difference. Voepel-Lewis et al. reported a minimum clinically important difference of −1 (95% −0.5 to 1) or + 1 (95% 0.5 to 2.7) indicating feeling “a little better” or “worse” in children with postoperative pain [20]. The results of our study indicate that the difference in pain scores, which was observed to be between 2 and points on an 11-point scale, was clinically relevant. We chose the first postoperative day as the primary outcome because this period is when patients typically experience the most intense pain following surgery, which then gradually decreases over the next few days [21].

We were unable to find studies comparing the different analgesia techniques in pediatric patients undergoing initial major abdominal surgery for intractable functional constipation specifically. Regrettably, there exists a paucity of contemporary literature on this subject. The guidelines regarding postoperative pain in children encounter a similar limitation [22,23]. However, few studies have reported on pain in children undergoing other abdominal surgeries with different analgesic techniques. First, a systematic review by Baeriswyl et al. was conducted for 195 pediatric patients undergoing any type of abdominal surgery [24]. In this study, the effectiveness of a transversus abdominus plane block was compared to epidural or caudal analgesia, with pain on the first postoperative day as the primary outcome. After a meta-analysis was performed, both pain control methods appeared equally effective. This contradicts our findings of superior analgesic effects with neuraxial analgesia, which could possibly be explained by the difference in indication for surgery. Baeriswyl et al. investigated any abdominal surgery, while we specifically studied abdominal surgeries for intractable functional constipation. Our more “complex” patient group may experience higher levels of postoperative pain, making it more likely to benefit from neuraxial analgesia as compared to those with less severe pain. Another explanation could be the difference in age of the surgical population. Baeriswyl et al.’s patients were between 1 and 9 years of age, while our patients had a median age of 13.5 years. It is possible that sensitization plays a greater role in older patients, and as a result, the prolonged analgesic effect of an epidural compared to a truncal block has a greater advantage in older children.

Two RCTs reported on the effectiveness of different analgesic techniques, both in patients undergoing lower abdominal surgery, such as hernia repair and orchidopexy. Ipek et al. conducted a RCT with 94 pediatric patients between 6 months and 14 years. The patients were randomly divided into three equal groups with either a transversus abdominis plane block, quadratus lumborum block, or caudal epidural block performed [25]. This study reported a lower pediatric objective pain scale (POAS) score in patients with a quadratus lumborum block at the second and fourth hour after surgery. Sethi et al. conducted a RCT including 80 children between 2 and 6 years who were allocated to either a transversus abdominis plane block or a caudal/epidural block group [26]. After surgery, pain was assed using the Face, Legs, Activity, Cry and Consolability (FLACC) scale. This study reported similar pain scores in both groups from 10 min to 24 h after surgery. The result of both RCTs indicated that neuraxial analgesia did not provide superior pain relief. Our hypothesis is that this may be attributed to the less complex patient population as well as the less extensive nature of the procedure, as previously discussed.

Mansfield et al. conducted a retrospective study involving 186 pediatric patients undergoing laparotomy, with 151 patients receiving epidural analgesia and 35 without epidural analgesia. The mean pain scores were significantly lower solely on the day of surgery in patients who received epidural analgesia compared to those without. However, the pain intensity exhibited a comparable pattern between both groups during the initial three days following the surgical procedure [27].

In our study, we did not find a difference in the length of stay in the PACU or overall hospitalization time. This finding is in contrast to the results reported by Baeriswyl et al., who found that patients undergoing any type of abdominal surgery and receiving a transversus abdominis block had a shorter length of hospital stay (MD −0.6, 95% CI −0.9 to −0.3 days) compared to those patients with epidural analgesia [24]. However, it is important to note that in our patient group, the length of stay was likely determined more by other factors, such as the management of function of the surgical procedure, rather than by pain management alone. Nonetheless, adequate pain management is still crucial for ensuring the quality of care for these patients [28].

This study has several limitations. Because of the retrospective design, the outcomes could have been exposed to various kinds of bias. The main disadvantage is confounding by indication. Patients with a more complicated and longer laparoscopic surgery could have received more likely a truncal block, whereby the worse baseline condition might explain the higher pain scores in this specific group. Another limitation is the fact that patients were recruited over a period of 11 years. This long recruitment period may introduce variability into the patient management, anesthesia protocols, and surgical approaches, which can affect the homogeneity and relevance of the study population. It may also limit the generalizability of the findings to the current medical practices and standards of care. Another disadvantage was the missing data. Five patients were excluded from this study because of missing data, which could bias the results. Lastly, patient-reported outcome measurements, such as satisfaction, are missing, which might have proved or disproved the clinical importance of the observed differences [29]. However, with a retrospective cohort study, such biases cannot be excluded. Nevertheless, the results obtained are valuable for hypothesis formation.

To further enhance understanding, we suggest the establishment of a global database encompassing pediatric cases with intractable functional constipation undergoing surgery. Only through this approach can we accrue substantial data for robust conclusions. Additionally, advocating for the utilization of Patient-Reported Outcome Measures (PROMs) and Patient-Reported Experience Measures (PREMs) over pain intensity scoring methods such as the NRS score and Visual Analogue Scale (VAS) is recommended for pain evaluation. PROMs and PREMs offer a comprehensive assessment, extending beyond mere pain intensity quantification to encompass functional limitations, emotional well-being, and the holistic patient experience with pain [30]. Evaluating the long-term outcomes rather than focusing solely on hospitalization periods is also advisable. Lastly, implementing strategies such as a Transitional Pain Service (TPS) might prove beneficial for this specific patient cohort [31]. A TPS refers to a specialized program designed to address pain management needs during the transition from hospital discharge to the home setting or to ongoing care. This service focuses on providing comprehensive pain management strategies and support for patients who have undergone surgery or experienced significant pain-related issues.

## 5. Conclusions

In summary, based on our findings, it can be hypothesized that neuraxial analgesia is the most advantageous pain management technique for pediatric patients undergoing initial major abdominal surgery for intractable functional constipation. However, due to the small sample size of our study, it is difficult to make definitive conclusions regarding rare but potentially serious complications. Therefore, well-designed studies with rigorous methodology to comprehensively evaluate the optimal pain management strategy for this unique patient group are warranted.

## Figures and Tables

**Table 1:** Patient characteristics.

	*N* = 65
Female sex	46 (70.8%)
Age at first surgery, years †	13.5 [8.8, 16.1]
BMI at first surgery, kg/m^2^ †	18.3 [15.8, 21.3]
Age of onset constipation, years †	2.0 [0, 7.0]
Age of first contact with tertiary hospital, years †	9.1 [5.9, 14.0]
sychological history	
PTSD	4 (6.2%)
Anxiety disorder	8 (12.3%)
Depression	2 (3.1%)
Behavioral problems	
Autism/PDD-NOS	10 (15.4%)
ADHD/ADD	5 (7.7%)
Eating disorder	2 (3.1%)
Developmental delay	8 (12.3%)
Previous surgeries	
Cecostomy button	22 (30.6%)
PEG tube	21 (32.8%)
Appendicostomy	3 (4.6%)

Variable distributions were reported as numbers and percentages unless specified otherwise. † Median [IQR]. Abbreviations: BMI = body mass index, PTSD = post-traumatic stress disorder, PDD-NOS = pervasive development disorder not otherwise specified, ADHD = attention deficit hyperactivity disorder, ADD = attention deficit disorder, PEG = percutaneous endoscopic gastrostomy.

**Table 2:** Perioperative characteristics.

	Systemic Analgesia (*n* = 43)	Neuraxial Analgesia (*n* = 17)	Truncal Block (*n* = 5)
Age at first surgery, years †	13.5 [8.9, 16.2]	11.3 [4.8, 14.0]	15.9 [15.8, 17.6]
Type of procedure ‡			
Ileostomy	29 (67.4%)	7 (41.2%)	4 (80.0%)
Colostomy	9 (20.9%)	3 (17.6%)	-
Sigmoidectomy	6 (14.0%)	8 (47.1%)	1 (20.0%)
Subtotal colectomy	1 (2.3%)	3 (17.6%)	1 (20.0%)
Technique			
Laparoscopic	36 (83.7%)	6 (35.3%)	3 (60.0%)
Open	4 (9.3%)	4 (23.5%)	-
Hand-assisted	3 (7.0%)	7 (41.2%)	2 (40.0%)
Duration surgery, minutes	122.0 [77.5, 145.5]	150.0 [135.0, 198.0]	202.0 [109.0, 220.0]
Analgesia intraoperative			
Paracetamol	15 (34.9%)	9 (52.9%)	1 (20.0%)
Metamizole	22 (51.2%)	9 (52.9%)	4 (80.0%)
Total opioids in IV MME †	29.0 [18.8, 50.0]	45.0 [12.5, 70.0]	40.0 [40.0, 47.0]
Total opioids in IV MME/kg †	0.4 [0.3, 0.6]	0.3 [0.3, 0.5]	0.2 [0.2, 0.4]
Clonidine or dexmedetomidine	7 (16.3%)	5 (29.4%)	3 (60.0%)
Esketamine continuous	5 (11.6%)	-	2 (40.0%)
Lidocaine continuous	3 (7.0%)	-	1 (20.0%)
Analgesia postoperative			
Paracetamol, orally	42 (97.7%)	15 (88.2%)	5 (100.0%)
NSAID, orally	18 (41.9%)	7 (41.2%)	2 (40.0%)
Metamizole, IV	25 (58.1%)	12 (63.2%)	4 (80.0%)
Tramadol, orally	8 (18.6%)	2 (11.8%)	-
Oxycodone, orally	9 (20.9%)	4 (23.5%)	3 (60.0%)
PCA opioids	13 (27.1%)	6 (31.6%)	1 (20.0%)
Duration PCA opioids, days †	3.0 [2.0, 4.0]	5.0 [4.0, 6.0]	6.0 [3.0, 6.0]
Duration strong opioids, days †	2.0 [1.0, 4.0]	0.0 [0.0, 3.0]	6.0 [5.0, 6.0]
Clonidine, orally	10 (23.3%)	6 (35.3%)	3 (60.0%)
Esketamine, IV	4 (9.3%)	2 (11.8%)	2 (40.0%)
Duration esketamine, day †	2.0 [2.0, 3.2]	5.0 [3.5, 6.5]	2.0 [2.0, 2.0]
Postoperative nausea and vomiting	2 (4.7%)	3 (17.6%)	3 (60.0%)

Variable distributions were reported as numbers and percentages (Fisher’s exact-test) unless specified otherwise. † Median and interquartile range. ‡ Procedures could be combined during surgery. Abbreviations: IV = intravenous, MME = morphine milligram equivalents, NSAID = non-steroidal anti-inflammatory drugs, PCA = patient-controlled analgesia.

**Table 3:** Differences in postoperative pain between three different analgesic techniques in patients with gastrointestinal motility disorder undergoing abdominal surgery.

	Systemic Analgesia (*n* = 43)	Neuraxial Analgesia (*n* = 17)	Truncal Block (*n* = 5)	*p*-Value
NRS 0–24 h (269 NRS scores) †,‡	5.0 [2.0, 7.0]	2.0 [0, 4.0]	5.0 [3.0, 6.5]	<0.001 ***
NRS 24–48 h (187 NRS scores) †,‡	4.0 [1.0, 6.0]	2.0 [0, 4.0]	4.0 [3.0, 6.5]	0.003 **
NRS 48–72 h (190 NRS scores) †,‡	4.5 [2.0, 6.0]	2.0 [0, 5.0]	5.0 [4.0, 6.8]	0.009 **
NRS 72–96 h (136 NRS scores)	3.5 [0, 5.0]	3.0 [0, 5.0]	5.0 [3.0, 6.0]	0.117
NRS 96–120 h (120 NRS scores)	4.0 [1.0, 6.0]	2.0 [0, 5.0]	4.0 [2.0, 8.0]	0.250

Variable distributions were reported as medians and interquartile range (Kruskal–Wallis test) unless specified otherwise. *: *p* < 0.05, **: *p* < 0.01, ***: *p* < 0.001. If the Kruskal–Wallis test was significant, the Dunn test was performed to determine which groups differed from each other. † Significant difference between neuraxial vs. systemic. ‡ Significant difference between neuraxial vs. truncal block. Abbreviations: NRS = numeric rating scale.

**Figure A1:**
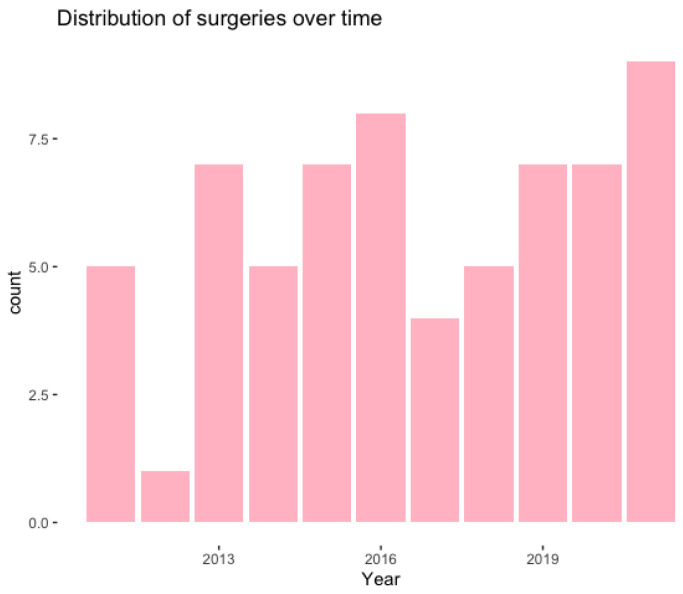
Distribution of surgeries over time.

## Data Availability

The data that support the findings of this study are available from the corresponding author upon reasonable request. This includes de-identified participant data, a data dictionary, and the informed consent form. These data will be available after the last publication for up to 10 years thereafter.
